# Gut mycobiota alterations in patients with COVID-19 and H1N1 infections and their associations with clinical features

**DOI:** 10.1038/s42003-021-02036-x

**Published:** 2021-04-13

**Authors:** Longxian Lv, Silan Gu, Huiyong Jiang, Ren Yan, Yanfei Chen, Yunbo Chen, Rui Luo, Chenjie Huang, Haifeng Lu, Beiwen Zheng, Hua Zhang, Jiafeng Xia, Lingling Tang, Guoping Sheng, Lanjuan Li

**Affiliations:** 1 State Key Laboratory for the Diagnosis and Treatment of Infectious Diseases, National Clinical Research Center for Infectious Diseases, Collaborative Innovation Center for Diagnosis and Treatment of Infectious Diseases, The First Affiliated Hospital, College of Medicine, Zhejiang University, Hangzhou, China; 2 Department of Infectious Diseases, Shulan (Hangzhou) Hospital, Affiliated to Shulan International Medical College, Zhejiang Shuren University, Hangzhou, China

**Keywords:** Infectious diseases, Dysbiosis, Fungal ecology

## Abstract

The relationship between gut microbes and COVID-19 or H1N1 infections is not fully understood. Here, we compared the gut mycobiota of 67 COVID-19 patients, 35 H1N1-infected patients and 48 healthy controls (HCs) using internal transcribed spacer (ITS) 3-ITS4 sequencing and analysed their associations with clinical features and the bacterial microbiota. Compared to HCs, the fungal burden was higher. Fungal mycobiota dysbiosis in both COVID-19 and H1N1-infected patients was mainly characterized by the depletion of fungi such as *Aspergillus* and *Penicillium*, but several fungi, including *Candida glabrata*, were enriched in H1N1-infected patients. The gut mycobiota profiles in COVID-19 patients with mild and severe symptoms were similar. Hospitalization had no apparent additional effects. In COVID-19 patients, Mucoromycota was positively correlated with *Fusicatenibacter*, *Aspergillus niger* was positively correlated with diarrhoea, and *Penicillium citrinum* was negatively correlated with C-reactive protein (CRP). In H1N1-infected patients, *Aspergillus penicilloides* was positively correlated with Lachnospiraceae members, *Aspergillus* was positively correlated with CRP, and Mucoromycota was negatively correlated with procalcitonin. Therefore, gut mycobiota dysbiosis occurs in both COVID-19 patients and H1N1-infected patients and does not improve until the patients are discharged and no longer require medical attention.

## Abstract (web summary)

Lv et al. associate the gut mycobiota with clinical features and the bacterial microbiota by comparing COVID-19 patients to those infected with H1N1 and healthy controls. They find that gut mycobiota dysbiosis occurs in both COVID-19 patients and those infected with H1N1 and that it does not improve until patients no longer require medical attention, providing insights into a better healthcare guideline.

## Introduction

SARS-CoV-2 has much higher infectivity and is associated with a much higher mortality rate than influenza viruses^1,2^. Meanwhile, the treatment of coronavirus disease 2019 (COVID-19) caused by SARS-CoV-2 is quite different from that of influenza caused by influenza viruses such as influenza A (H1N1). However, the early symptoms of COVID-19, including fever, cough, vomiting, and diarrhoea, are very similar to those of influenza^3,4^. Therefore, this will pose greater challenges for the prevention and treatment of COVID-19 when flu season arrives.

The normal mycobiota, particularly in the gut, plays important roles in host immune homeostasis, metabolism and infection prevention^5,6^. In contrast, gut fungal dysbiosis and fungal infections are related to a large number of diseases, such as inflammatory bowel disease, colorectal cancer, and asthma^7–11^. The development of some infectious diseases is also closely associated with gut fungi, such as the potential link between the enrichment of enteric fungi and hepatitis B virus infection^12^. Furthermore, some fungi, such as *Candida albicans*, are able to decrease host susceptibility to colitis and H1N1 virus infection^13^.

The rate of fungal co-infection in COVID-19 patients is significantly higher in those with severe cases than in those with mild cases^14^; this is also observed in H1N1-infected patients. Moreover, fungal co-infection strongly affects the prognosis of H1N1-infected patients^15–17^. H1N1 virus infection causes an impaired host immune response to fungi, which in turn leads to fungal proliferation^18^. However, it is still unclear whether alterations in the gut mycobiota occur and whether they are associated with the health status of patients with COVID-19 and those infected with H1N1. This work aims to explore the alterations in clinical characteristics, gastrointestinal symptoms, inflammation, and structure of the gut mycobiota in COVID-19 patients and H1N1-infected patients compared with healthy individuals. Furthermore, the associations of an altered gut mycobiota with the gut microbiota and clinical features were explored.

## Results

### Clinical symptoms were similar between H1N1-infected patients and COVID-19 patients

Nearly 54% of COVID-19 cases and 71% of H1N1 cases were severe (Table 1). The most common underlying diseases were hypertension, diabetes mellitus, heart disease, and liver diseases, while the most common symptoms were fever, cough and diarrhoea in both COVID-19 patients and H1N1-infected patients, although these factors were not significantly different between the two groups (Table 1). The rate of positivity for SARS-CoV-2 RNA in the faeces of COVID-19 patients was 34.32%. All patients with H1N1 infection or COVID-19 were treated with antiviral drugs; some were treated with glucocorticoids. The hospital stays were longer for COVID-19 patients than for H1N1-infected patients.

**Table 1:** Baseline characteristics and clinical course.

	COVID-19 (*n* = 67)	H1N1 (*n* = 35)	Healthy controls (*n* = 48)	*P*-value (COVID-19 versus H1N1)
Median (interquartile range) age (years)	52.0 (42.0, 60.0)	53.0 (43.0, 62.0)	59.0 (40.0, 78.0)	0.094
Male sex, no./total (%)	43 (46.27%)	24 (68.00%)	25 (52.08%)	0.889
BMI, kg/m^2^	23.6 (22.2, 24.7)	24.3 (22.1, 26.4)	21.8 (24.0, 26.3)	0.505
Severe, no./total (%)	36 (53.73%)	25 (71.43%)	–	0.085
Underlying diseases				
Hypertension	16 (23.88%)***	3 (8.57%)*	0	0.067
Diabetes mellitus	10 (14.92%)**	2 (5.71%)	0	0.173
Heart disease	4 (5.97%)	0	0	0.142
Liver diseases	2 (2.99%)	3 (8.57%)*	0	0.217
Symptoms				
Fever	61 (91.04%)***	31 (91.43%)***	0	0.691
Cough	54 (80.59%)***	33 (95.71%)***	0	0.065
Diarrhoea	7 (10.44%)*	4 (11.43%)*	0	0.880
Vomiting	5 (7.46%)	5 (14.28%)**	0	0.274
Abdominal pain	2 (3.08%)	2 (3.08%)	0	0.502
Stool with SARS-CoV-2	23 (34.32%)	–	0	–
Medication use				
Antivirals	67 (100%)	35 (100%)	–	1.00
Glucocorticoids	49 (73.13%)	10 (28.57%)	–	0.001
Outcomes				
Mortality	0	0	0	1.00
Median (interquartile range) of hospital days	16 (13, 22)	7 (6, 9)	0	9.67E−07

*BMI* body mass index.

*P < 0.05; **P < 0.01; and ***P < 0.001 when compared with the healthy controls.

### Different alterations in inflammation and organ function between H1N1-infected patients and COVID-19 patients

**Fig. 1:**
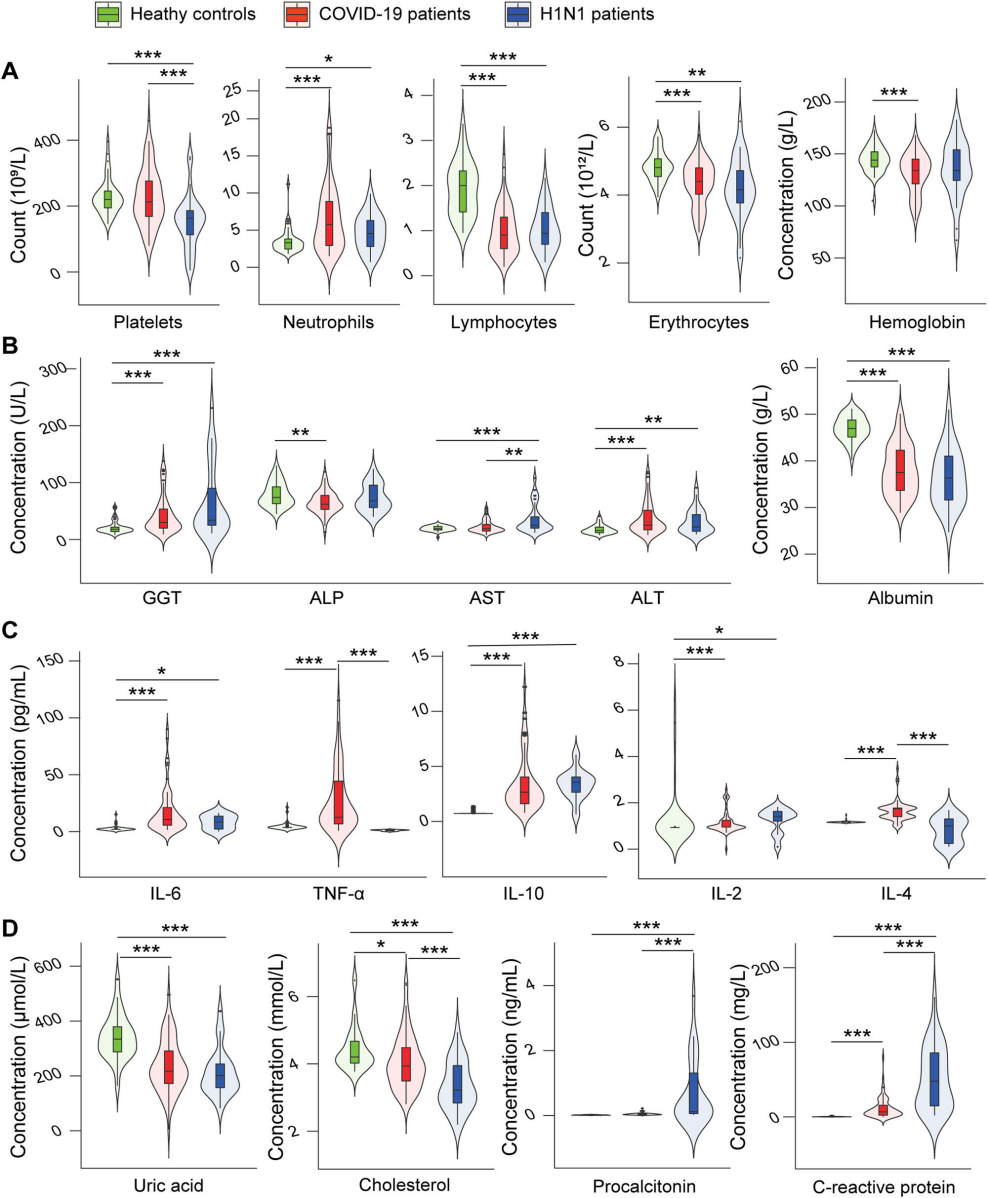
Alterations in inflammatory, hepatic, metabolic and infectious biomarkers in blood from patients with COVID-19 or H1N1 infections compared with HCs. Significantly altered (**A**) blood haemocytes and haemoglobin, **B** liver function indicators, **C** inflammatory cytokines, and (**D**) metabolic and infectious biomarkers in the blood of patients with COVID-19 (*n* = 67) or H1N1 (*n* = 35) infections compared with HCs (*n* = 48). *P < 0.05; **P < 0.01; and ***P < 0.001.

Both COVID-19 patients and H1N1-infected patients had lower blood lymphocyte counts and higher neutrophil counts than HCs (Fig. 1A). The level of CRP, an index of bacterial infection, was significantly higher in COVID-19 patients and H1N1-infected patients than in HCs, while procalcitonin, an index of bacterial and fungal infection, was higher in H1N1-infected patients than in COVID-19 patients and HCs (Fig. 1D). Additionally, compared with HCs, both H1N1-infected patients and COVID-19 patients had significant increases in the levels of IL-2, IL-6, and IL-10, suggesting that the patients were in an inflammatory state (Fig. 1C). IL-4 and TNF-α levels were higher in COVID-19 patients than in H1N1-infected patients and HCs, suggesting that COVID-19 patients experienced more inflammation. Compared with HCs, the level of albumin was lower and the levels of ALT and GGT were higher in both H1N1-infected patients and COVID-19 patients, suggesting that the patients experienced liver injury. Interestingly, AST was only higher in H1N1-infected patients and was even higher in these patients than in COVID-19 patients, suggesting that more serious liver injury might occur in H1N1-infected patients. ALP was only lower in COVID-19 patients than in HCs (Fig. 1B). Compared with the HCs, the absolute erythrocyte count in the peripheral blood was lower in both COVID-19 patients and H1N1-infected patients, while the level of haemoglobin was only lower in the COVID-19 group, suggesting that H1N1 infection, especially COVID-19, may cause erythrocyte damage (Fig. 1A). In addition, compared with the HCs, the levels of cholesterol and uric acid were lower in COVID-19 and H1N1-infected patients, while the platelet count was only lower in H1N1-infected patients. Furthermore, the platelet count and cholesterol level were lower in H1N1-infected patients than in COVID-19 patients (Fig. 1A, D).

### Fungal richness decreased in the gut of H1N1-infected patients and COVID-19 patients

**Fig. 2:**
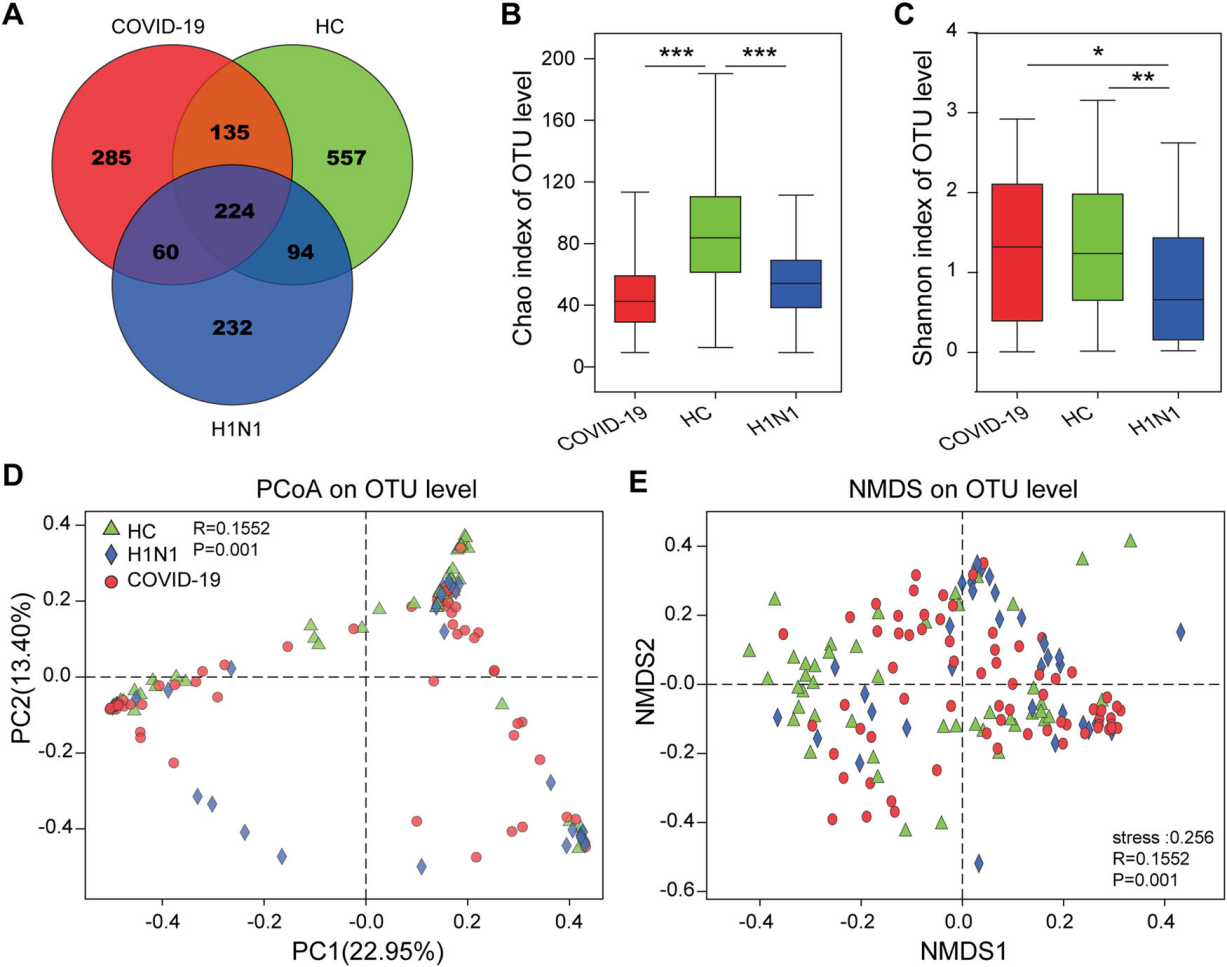
Alterations in gut fungal diversity in patients with COVID-19 or H1N1 infections compared with HCs. **A** Venn diagram, **B** Chao 1 index plot, **C** Shannon index plot, **D** PCoA plot, and (**E**) NMDS plot based on the gut fungal operational taxonomic units (OTUs) of COVID-19 patients (*n* = 67), H1N1-infected patients (*n* = 35) and HCs (*n* = 48). *P < 0.05; **P < 0.01; and ***P < 0.001.

Through fungal ITS sequencing, 1587 OTUs were identified, including 557 OTUs unique to HCs, 232 OTUs unique to H1N1-infected patients, and 285 OTUs unique to COVID-19 patients (Fig. 2A). The OTU richness, as reflected by the Chao1 index, was not significantly different between COVID-19 patients and H1N1-infected patients, but that in each patient group was lower than that in the HCs (Fig. 2B). The OTU diversity, as measured by the Shannon index, was similar between COVID-19 patients and HCs and was higher in both groups than in the patient group infected with H1N1 (Fig. 2C). In the PCoA plots, HCs, COVID-19 patients and H1N1-infected patients were clustered separately (Fig. 2D), indicating that their compositions were significantly different; similar results were observed in non-metric multi-dimensional scaling (NMDS) plots (Fig. 2E). These conclusions were also confirmed by permutational multivariate analysis of variance (*P* = 0.001).

### Fungal dysbiosis in the guts of H1N1-infected patients and COVID-19 patients

**Fig. 3:**
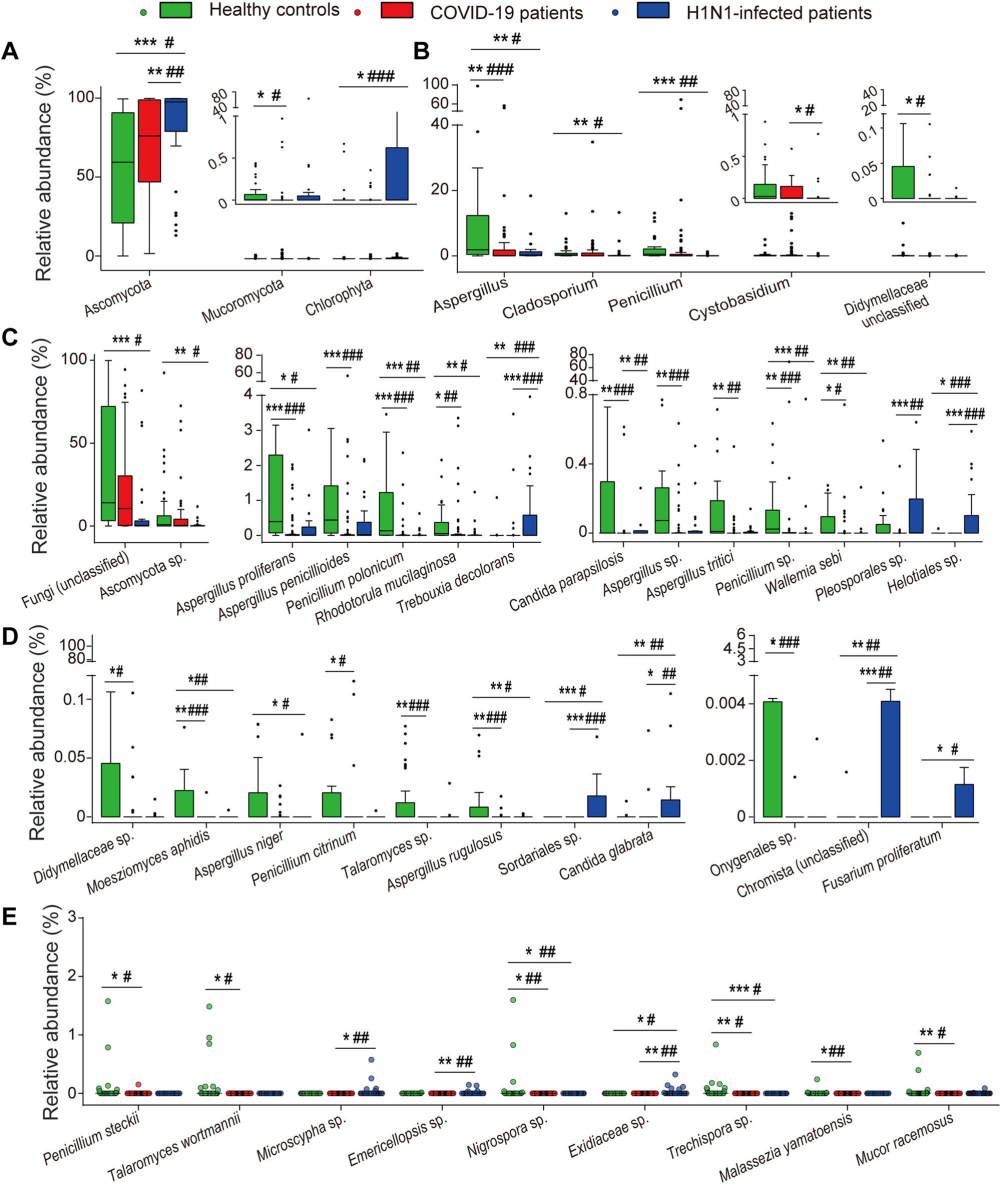
Alterations in the gut fungal taxa in patients with COVID-19 or H1N1 infections compared with HCs. **A** Phyla, **B** genera, and (**C**–**E**) species that were differently distributed between at least two groups (COVID-19 patients, H1N1-infected patients and HCs) in both the discovery and validation cohorts. The figure was generated by combining data from the discovery and validation cohorts (COVID-19, *n* = 67; H1N1, *n* = 32; HCs, *n* = 46). *P < 0.05; **P < 0.01; and ***P < 0.001 in the discovery cohort. ^#^*P* < 0.05; ^##^*P* < 0.01; and ^###^*P* < 0.001 in the validation cohort.

Compared with HCs, all significant alterations in the gut mycobiota in COVID-19 patients were depletions of specific fungal taxa, such as members of Ascomycota and Basidiomycota, which was observed in both the discovery (COVID-19, *n* = 34; HCs, *n* = 23) and validation (COVID-19, *n* = 33; HCs, *n* = 23) cohorts. In the phylum Ascomycota, most of the depleted taxa belonged to *Aspergillaceae*, such as *Penicillium citrinum*, *Penicillium polonicum*, and *Aspergillus* with its five species (Fig. 3B–E). Furthermore, *Candida parapsilosis*, *Talaromyces wortmannii*, and two unclassified species that separately belonged to *Didymellaceae* or *Onygenales* were also depleted in COVID-19 patients. In the phylum Basidiomycota, five species, including *Malassezia yamatoensis*, *Rhodotorula mucilaginosa*, *Moesziomyces aphidis*, *Trechispora* sp. and *Wallemia sebi*, were significantly depleted (Fig. 3C–E). Similar results were observed in the phylum Mucoromycota and for the species *Mucor racemosus* (Fig. 3A–E). Fungal taxa, such as *Candida albicans*, that were altered in COVID-19 patients versus HCs in only the discovery or validation cohorts are shown in Supplementary Fig. 1.

Compared with HCs, H1N1-infected patients were mainly characterized by enrichment of the phylum Ascomycota and depletion of an unclassified fungus, which was observed in both the discovery (H1N1, *n* = 20; HCs, *n* = 30) and validation (H1N1, *n* = 12; HCs, *n* = 16) cohorts (Fig. 3A). In the phylum Ascomycota, *Candida glabrata*, *Fusarium proliferatum*, and two species belonging to Helotiales and Sordariales were enriched; meanwhile, fungi such as *Cladosporium*, *Aspergillus*, *Penicillium*, *Aspergillus niger*, and *Penicillium polonicum* were depleted in H1N1-infected patients. In the phylum Basidiomycota, an unclassified species of *Exidiaceae* was enriched, while *Trechispora* sp., *Rhodotorula mucilaginosa*, *Moesziomyces aphidis*, and *Wallemia sebi* were depleted (Fig. 3B–E). Interestingly, the phylum Chlorophyta (belonging to the kingdom Plantae) and its species *Trebouxia decolorans* (Fig. 3A, C) as well as an unclassified species belonging to the kingdom Chromista were enriched in the gut mycobiota of H1N1-infected patients (Fig. 3D). Additionally, fungal taxa such as *Saccharomyces cerevisiae* that were altered in H1N1-infected patients compared with the HCs in only the discovery or validation cohorts are shown in Supplementary Fig. 2.

Compared with H1N1-infected patients, the alterations in the gut mycobiota of COVID-19 patients were mainly characterized by the depletion of the Ascomycota taxa, as observed in both the discovery (COVID-19, *n* = 34; H1N1, *n* = 20) and validation (COVID-19, *n* = 33; H1N1, *n* = 12) cohorts (Fig. 3A). The phylum Ascomycota, as well as its members *Candida glabrata* and *Candida parapsilosis*, and five unclassified species separately belonging to Helotiales, Pleosporales, Sordariales, *Microscypha* or *Emericellopsis* were depleted in COVID-19 patients (Fig. 3C–E). In the phylum Basidiomycota, *Cystobasidium* was enriched, while an unclassified species of *Exidiaceae* was depleted. Moreover, *Trebouxia decolorans* and an unclassified species belonging to the kingdom Chromista were also depleted in COVID-19 patients compared to H1N1-infected patients (Fig. 3C–E). Additionally, fungal taxa such as *Meyerozyma guilliermondii* that were altered in COVID-19 patients compared with H1N1-infected patients in only the discovery or validation cohorts are shown in Supplementary Fig. 3.

The *P* values of ANOSIM comparing the gut mycobiota compositions of patients with mild and severe COVID-19 (*P* = 0.69) and comparing those of COVID-19 patients in and out of the hospital (*P* = 0.93) were both greater than 0.05. This indicates that the composition of the gut mycobiota was similar in these groups and was not significantly influenced by COVID-19 severity or treatment in our cohorts.

In the receiver operating characteristic (ROC) curve analysis, when the area under the ROC curve (AUC) of both the discovery and validation cohorts higher than 0.7 (which is indicative of a discriminatory effect) was taken as a threshold, no signal fungal taxa could reliably discriminate COVID-19 patients from HCs or H1N1-infected patients. In contrast, *Trebouxia decolorans* could discriminate H1N1-infected patients from HCs or COVID-19 patients (Supplementary Fig. 4A). Remarkably, only *Penicillium polonicum* could discriminate HCs from both H1N1-infected patients and COVID-19 patients, suggesting that this species is a potential health marker of the gut mycobiota (Supplementary Fig. 4B).

### Fungal burden increased in the guts of H1N1-infected patients and COVID-19 patients

**Fig. 4:**
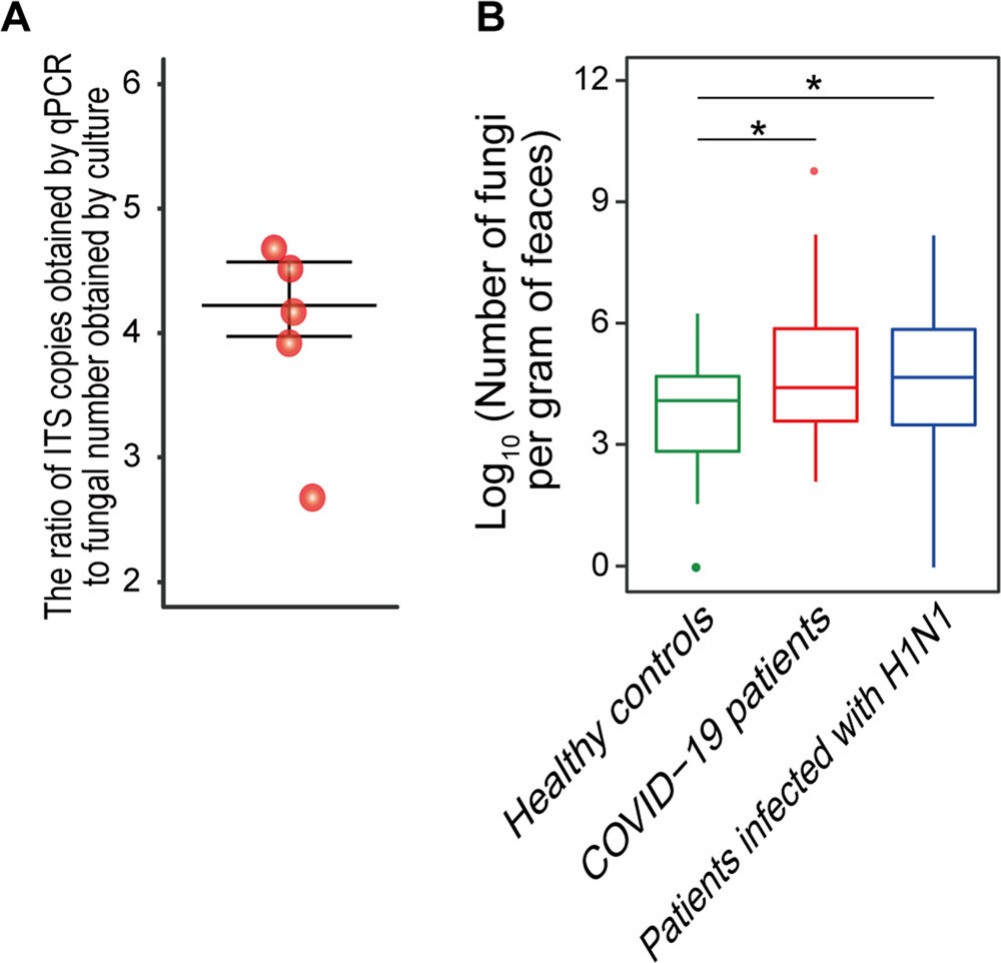
Fungal burden increased in the gut of H1N1-infected patients and COVID-19 patients. **A** The ratio of ITS copies of *Clavispora lusitaniae* CICC 32908 obtained by qPCR to CICC 32908 number obtained by culture. The experiment was repeated five times. **B** Number of fungi in one gram of faeces from the healthy controls (*n* = 48), COVID-19 patients (*n* = 67) or H1N1-infected patients (*n* = 35). The ITS copies in the faeces obtained by qPCR were converted to fungal numbers based on the ratio of ITS copies of *Clavispora lusitaniae* CICC 32908 obtained by qPCR to CICC 32908 number obtained by culture. *P < 0.05; **P < 0.01; and ***P < 0.001.

The total quantity of fungi in H1N1-infected patients, COVID-19 patients or HCs was assayed by qPCR. First, we evaluated the ratio of ITS copies of fungi obtained by qPCR to the amount of fungi obtained by plate counting using *Clavispora lusitaniae* CICC 32908 as a reference strain. Our results showed that this ratio was 4.04 ± 0.56 (Fig. 4A). Next, we performed qPCR using the DNA extract of faecal samples. The median (25th, 75th centiles) number of fungi per gram of faeces was 2.73E+04 (3.81E+03, 9.70E+05) for COVID-19 patients, 4.93E+04 (1.72E+03, 1.35E+06) for H1N1-infected patients, and 1.29E+04 (6.03E+02, 5.43E+04) for HCs. Although it is limiting to use *Clavispora lusitaniae* CICC 32908 to evaluate the abovementioned ratio, the total amount of fungi in the faeces of either COVID-19 patients or H1N1-infected patients was significantly higher than that in HCs in this study with the same method (Fig. 4B). In addition, we found no significant difference in the number of gut fungi between COVID-19 patients at admission and discharge.

### Associations between faecal mycobiota and the bacterial microbiota

**Fig. 5:**
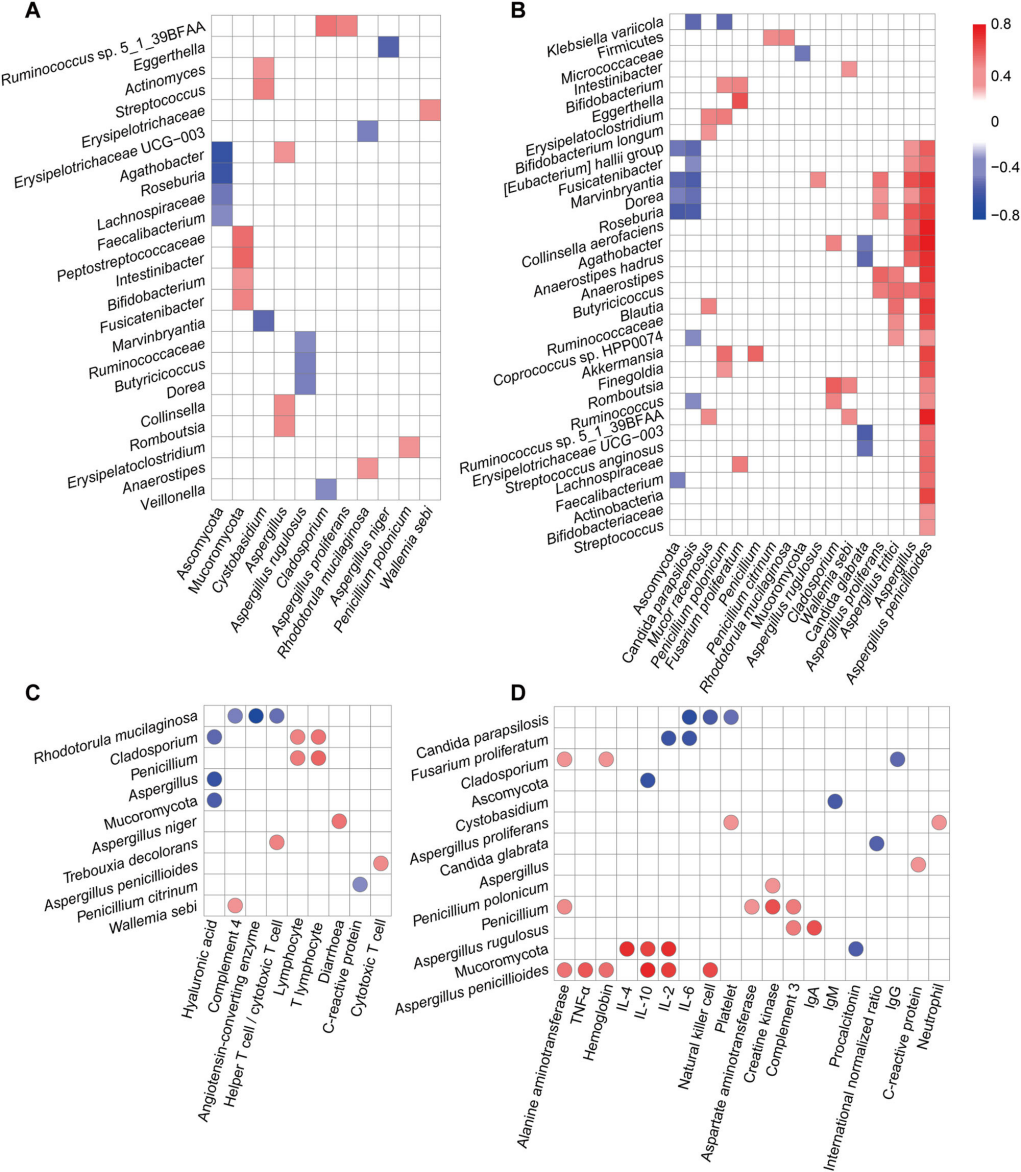
Altered gut fungal taxa are strongly linked with gut bacterial taxa and blood inflammatory, liver, infectious and metabolic biomarkers. Spearman correlations of significantly altered gut mycobiota with significantly altered gut microbiota in (**A**) COVID-19 patients or (**B**) H1N1-infected patients. Spearman correlations of significantly altered gut mycobiota with significantly altered blood biomarkers in (**C**) COVID-19 patients or (**D**) H1N1-infected patients. Only correlations with a *P* < 0.05 and a correlation coefficient >0.4 or <−0.4 are shown.

In COVID-19 patients, the phylum Ascomycota and its members *Aspergillus niger* and *Aspergillus rugulosus* were negatively correlated with *Lachnospiraceae* and its genera *Agathobacter*, *Dorea* and *Roseburia*; with *Ruminococcaceae* and its genera *Butyricicoccus* and *Faecalibacterium*; and with *Eggerthella* and *Veillonella* (Fig. 5A). In contrast, the phylum Mucoromycota was positively correlated with *Peptostreptococcaceae*, *Bifidobacterium*, *Fusicatenibacter* and *Intestinibacter*, as well as *Aspergillus* with *Agathobacter*.

In H1N1-infected patients, more correlations between the gut mycobiota and microbiota were observed, and the absolute values of their correlation coefficients were higher as well (Fig. 5B). More than half of these correlations were positive for *Aspergillus* and its genera with *Lachnospiraceae*, *Ruminococcaceae*, and *Erysipelotrichaceae*, as well as their members. Interestingly, *Penicillium* and *Penicillium polonicum* were also positively correlated with *Akkermansia*. In contrast, there were negative correlations of Ascomycota with bacteria such as *Roseburia* or *Marvinbryantia*.

### Associations of the gut mycobiota with clinical symptoms of H1N1 infection or COVID-19

In COVID-19 patients, *Aspergillus niger* was highly significantly positively correlated with the incidence of diarrhoea (*r* = 0.51, *P* = 8.31E−06), while *Penicillium citrinum* was highly significantly negatively correlated with the blood CRP level (r = −0.41, *P* = 9.37E−04) (Fig. 5C). Interestingly, *Rhodotorula mucilaginosa* was highly negatively correlated with blood angiotensin-converting enzyme (*r* = −0.79, *P* = 0.036). Furthermore, many gut fungi were positively correlated with blood immune indicators, such as the absolute number of blood lymphocytes. Remarkably, the faecal positivity rate of SARS-CoV-2 RNA was positively correlated with diarrhoea and disease severity.

In H1N1-infected patients, *Aspergillus* was positively correlated with the CRP level, while Mucoromycota was negatively correlated with the procalcitonin level (Fig. 5D). Furthermore, there were other positive correlations, such as those of *Aspergillus penicillioides*, with the serum levels of TNF-α, IL-2 and IL-10.

## Discussion

As an important component of human immunity and metabolism, gut microbes have attracted extensive attention in COVID-19^19–23^. In a pilot study of 15 patients with COVID-19, Zuo et al.^20^ found persistent alterations in the faecal microbiome during hospitalization compared with controls, and these alterations were associated with faecal levels of SARS-CoV-2 and COVID-19 severity. Furthermore, these authors reported heterogeneous configurations of the faecal mycobiome during hospitalization in 30 patients with COVID-19 compared with the controls^21^. Gu et al.^19^ observed that the gut microbial signature of patients with COVID-19 was different from those of H1N1-infected patients and HCs. In this work, we explored the differences in the gut mycobiota between patients with COVID-19 and those infected with H1N1, as well as between each group of patients and HCs, and analysed the associations of the altered gut mycobiota with the microbiota and clinical symptoms. All of the above studies contributed very important information about the pathogenesis, diagnosis and treatment of COVID-19.

Gastrointestinal symptoms such as diarrhoea, vomiting, and abdominal pain are frequently observed in patients with COVID-19 or H1N1. The proportion of our COVID-19 patients with gastrointestinal symptoms was 20.89%, which is within the range from 16.1 to 33.4% reported in studies from China and other countries^24,25^. Meanwhile, gastrointestinal symptoms were observed in 31.43% of our H1N1-infected patients, which was similar to the 30.9% in patients infected with A(H1N1)pdm09 in a meta-analysis^26^. The faecal positivity rate of SARS-CoV-2 RNA in our samples was 34.32%, and the rate ranged from 15.3 to 59% in previous reports^27^. Although we did not measure the rate of positivity for H1N1 viruses in faeces, a meta-analysis showed that it was 20.6%^24,25^. Furthermore, we found that the faecal positivity rate for SARS-CoV-2 RNA was positively correlated with diarrhoea and disease severity, indicating the potential impact of SARS-CoV-2 in the gut on gastrointestinal symptoms and disease severity.

Our results showed that both the fungal α-diversity and the relative abundance of the most altered taxa in the gut mycobiome of COVID-19 patients and H1N1-infected patients were significantly lower than those in HCs. Gut mycobiota homeostasis plays an important role in maintaining host immune and metabolic functions and has attracted increasing attention. In general, *Candida*, *Malassezia*, *Aspergillus*, *Epicoccum*, *Saccharomyces*, *Alternaria*, and *Cladosporium* are the most common gut fungi^5^. The long-term colonization of symbiotic fungi such as *Candida albicans* in the gut stimulates the proliferation of systemic fungal-specific Th17 cells and IL-17 feedback through neutrophil circulation in the blood, which can help the host fight infections by exogenous pathogens^28^. The destruction of lung immunity by antifungal agents, such as fluconazole, aggravated allergic airway disease in a mouse model when it reduced gut fungi^29^. These results are consistent with our findings showing that many depleted gut fungi are positively correlated with reductions in blood immune factors such as lymphocytes. Furthermore, symbiotic gut fungi can protect local and systemic immunity by providing complementary microbial stimulation in place of stimulation by bacteria. For example, after the eradication of symbiotic bacteria, the oral administration of *Candida albicans* or *Saccharomyces cerevisiae* can significantly protect mucosal tissue from damage, calibrate the responsiveness of circulating immune cells, and decrease host susceptibility to colitis and H1N1 virus infection. These protective effects are mainly a result of the gut stimulatory function of mannans, a highly conserved component of fungal cell walls^13^. Therefore, the depletion of commensal gut fungi in COVID-19 patients or H1N1-infected patients may lead to the loss of their beneficial functions^19^.

Our results show that some opportunistic pathogenic fungi were enriched in COVID-19 patients, and some of these fungi were particularly enriched in H1N1-infected patients compared to HCs. For example, of all *Candida* species, *Candida glabrata* accounts for approximately 10–35% of total *Candida* bloodstream infections^30^. *Cladosporium* species were reported to cause various superficial and invasive fungal infections, such as brain abscess and keratitis^31^. *Fusarium proliferatum* is a causative agent of human respiratory disorders; for example, this species caused pulmonary fusariosis in an immunocompetent diabetic patient with severe COVID-19^32^. Furthermore, *F. proliferatum* extracts induced a strong release of IL-8 in human lung epithelial cells (BEAS-2B)^33^. Undoubtedly, most of these fungi also live in the gut of some HCs. However, in the case of systemic inflammation, the gut barrier may be damaged, and the enrichment of these fungi increases the risk of infection. In fact, fungal infections were observed but were usually mild in severe COVID-19 or influenza patients^14,16^.

Our results showed that the gut fungal load in patients with COVID-19 or H1N1 infection was significantly higher than that in HCs. Similarly, a higher gut fungal load has been observed in patients with *Clostridium difficile* infection^34^, Crohn’s disease, ulcerative colitis^35^ or alcoholic liver disease^36^ than in healthy subjects. As reported in many studies, bacterial microbiota dysbiosis, extensive tissue damage and the presence of an inflammatory environment can cause fungal overgrowth in the gut. Conversely, the overgrowth of fungi such as *Candida albicans* or *Malassezia* exacerbated experimental colitis^37^; *Candida* overgrowth may contribute to inflammation in patients with alcoholic liver disease^38^. Therefore, as with many other diseases, it needs to be further clarified in the future whether the higher burden of fungi is a characteristic of these patients before infection by SARS-CoV-2 or H1N1 or the result of SARS-CoV-2 or H1N1 infection. If it is the former, the relationship between a higher fungal burden and susceptibility to SARS-CoV-2 or H1N1 infection and the underlying mechanism are worth exploring further. However, if it is the latter, the role and mechanism of fungal overgrowth with regard to the occurrence and development of SARS-CoV-2 or H1N1 infection, as well as the long-term impacts of fungal overgrowth on individuals after rehabilitation, is worth investigating. In addition, we found an increase in the total fungal abundance and a decrease in the relative abundance of some fungi in COVID-19 or H1N1 patients; thus, there is the possibility of a “bloom” of opportunistic pathogenic fungi, which likewise deserves a further attention in future studies.

Our results showed that some altered gut fungi in COVID-19 patients or H1N1-infected patients were closely associated with altered gut bacteria. Generally, the interactions between bacteria and fungi can be described as mutualism, commensalism or competition; these interactions can involve competing for the same food source or for the same space, secreting molecules to promote or inhibit growth or influence one another’s environment, and altering the host immune response^39,40^. For example, the culture supernatant of *Roseburia faecis* and *Roseburia intestinalis* had an inhibitory effect on the growth of several *Candida* spp. and *S. cerevisiae*, while that of *Bacteroides ovatu*s affected filamentation inhibition in vitro^41^. Furthermore, the cross-kingdom interactions between bacteria and fungi may have a major impact on the likelihood of mucosal and systemic infections and the severity of these infections^42^. For example, colonization with *Candida* spp. was reported to be associated with increased morbidity and a prolonged hospital stay in patients with ventilator-associated pneumonia^43^; disturbance in vaginal *Lactobacillus* abundance and *Candida* counts along with their interactions may lead to infections^44^; and some butyrate producers, such as *Agathobacter*, *Fusicatenibacter* and *Roseburia*, were depleted in COVID-19 patients and negatively correlated not only with Ascomycota but also with infectious markers such as CRP and procalcitonin^19^. Promisingly, many computationally inferred cross-domain interactions can be validated via coculture^45^, making the correlation results of bacteria and fungi more valuable.

In addition to being in the lung, SARS-CoV-2 and H1N1 receptors are also abundant in the gut, suggesting that viral infection and alterations in the mycobiota may be linked in the gut. Angiotensin I-converting enzyme 2 (ACE2), a receptor of both SARS-CoV and SARS-CoV-2, is a key regulator of the renin-angiotensin system (RAS), which is involved in acute lung failure, cardiovascular function and SARS^24,46^. Moreover, ACE2 can regulate intestinal amino acid homeostasis, the expression of antimicrobial peptides, and the ecology of gut microbes. The depletion of ACE2 in mice results in highly increased susceptibility to intestinal inflammation induced by epithelial damage. Conversely, transplantation of *Ace2* mutant mouse microbiota into germ-free wild-type mice can promote the propensity to develop severe colitis^47^. Importantly, SARS-CoV-2 RNA was detected in gastric, duodenal, and rectal epithelia by intracellular staining, demonstrating that SARS-CoV-2 infects these epithelial cells^48^. Similarly, it was reported that gastrointestinal epithelial cells were susceptible to H1N1 viruses using sialic acid (SA)-α2,6-galactose (Gal)-terminated saccharides as the receptor and became apoptotic after infection^49^. This observation indicates that gastrointestinal symptoms and alterations in the gut mycobiota during SARS-CoV-2 or H1N1 infections may form a pathogenic feedback loop, causing the disease to worsen.

The gut–lung axis may play a key role in linking alterations of the gut mycobiota and lung infection in SARS-CoV-2 and H1N1. The components and products of gut bacteria and fungi, such as lipopolysaccharides and short-chain fatty acids, which can be transported via the circulatory system, are important means of communication between the gut microbiota and the lungs. Furthermore, immune cells, such as intestinal group 2 innate lymphoid cells, cytokines and even hormones, can migrate from the gut to the respiratory system via the circulation^50^. Therefore, the depletion of gut fungi that have important functions and the enrichment of opportunistic pathogenic fungi may contribute to the development of pneumonia. In contrast, the lung can also affect intestinal health and gut mycobiota homeostasis. There is evidence that some pulmonary infections can directly affect gut immunity^51^. Therefore, in addition to direct viral infection in the gastrointestinal tract, pneumonia during SARS-CoV-2 or H1N1 infections may be one of the most important causes of gastrointestinal symptoms and alterations in the gut mycobiota.

Although this work attempts to provide comprehensive insights into the potential contributions of the gut mycobiota in COVID-19 and H1N1, there are several limitations to be addressed. First, we were not able to collect samples from asymptomatic patients infected by SARS-CoV-2. Their inclusion in future studies may help reveal the role of gut microbes in the pathogenicity of SARS-CoV-2. Second, the numbers of samples in the study, especially those of patients with typical gastrointestinal symptoms such as diarrhoea, were relatively small. This may affect the sufficiency of the association analysis results and is mainly because the proportion of patients with these symptoms is small. Third, using *Clavispora lusitaniae* CICC 32908 to evaluate the ratio of ITS copies of fungi obtained by qPCR to the amount of fungi obtained by plate counting has certain limitations, as there are many types of fungi in the gut, and the ratio of different fungi will be significantly different. Fourth, in addition to the fungal and bacterial flora we have explored in this work, there are also viral flora in the gut, which may also be very important in COVID-19, especially in patients with gastrointestinal infections^52^.

In summary, although many clinical symptoms are similar between COVID-19 patients and H1N1-infected patients, COVID-19 patients have some unique clinical features. For example, the levels of IL-4 and TNF-α were higher in COVID-19 patients than in H1N1-infected patients and HCs; the serum ALP and haemoglobin levels were specifically lower in COVID-19 patients than in HCs. The gut fungal burden was higher in H1N1-infected patients or SARS-CoV-2 than in the HCs; the relative abundances of some fungi with important functions were lower, but those of several opportunistic pathogenic fungi were higher in patients with COVID-19 or H1N1 infections than in the HCs; some Ascomycota taxa were depleted in the gut of COVID-19 patients compared with H1N1-infected patients; and the altered gut fungi were strongly associated with the gut microbiota and clinical characteristics such diarrhoea and procalcitonin level. The gut mycobiota were not different between COVID-19 patients with mild and severe symptoms or between COVID-19 patients in and out of the hospital. These results provide a perspective for understanding the clinical features, pathogenesis, and comprehensive diagnosis and treatment of COVID-9 and H1N1 infection.

## Methods

### Study design and sample collation

This study was approved by the ethics committee of the First Affiliated Hospital, Zhejiang University (IIT2020-136 and 2018-447) and all participants signed informed consent forms. SARS-CoV-2 or H1N1 infections were diagnosed by reverse-transcriptase polymerase chain-reaction assay using respiratory tract samples. The participating COVID-19 patients were admitted to the First Affiliated Hospital, Zhejiang University from January to March 2020. Additionally, H1N1-infected patients hospitalized from January 2018 to January 2020 were enrolled. All subjects who received antifungals, probiotic treatment or both within 4 weeks before enrolment were excluded.

A cohort of 150 subjects was recruited, including 67 COVID-19 patients, 35 H1N1-infected patients, and 48 age-, sex-, and BMI-matched healthy controls (HCs). The degree of severity of H1N1 infection (severe vs. non-severe) at the time of admission was defined using the WHO surveillance case definitions for severe acute respiratory infection (SARI)^53^. Mild and moderate cases of COVID-19 were grouped as non-severe, and severe and critical cases were grouped as severe in this work using the diagnostic criteria of the seventh edition of the Diagnostic and Treatment Protocol for COVID-19 in China^54^. The patient clinical characteristics, including demographic characteristics, comorbidities, symptoms, and clinical outcomes, were collected from the medical records.

Blood and faecal samples were collected when the COVID-19 patients and H1N1-infected patients were initially admitted to the hospital, as well as when the COVID-19 patients were discharged. Samples were stored at −80 °C until use.

### Analysis of haematological variables; liver, kidney and heart function; and serum cytokines

Blood samples were used to measure haematological indices and biochemical values using automated equipment and standard methods. Haematological variables, such as the counts of lymphocytes, neutrophils, and erythrocytes, were tested using a Sysmex XE-2100 system (Sysmex Corporation, Kobe, Japan). Indicators of liver function, including albumin (ALB), alkaline phosphatase (ALP), aspartate aminotransferase (AST), alanine aminotransferase (ALT), and glutamyl transpeptidase (GGT) levels, were assessed by standard methods using a 7600–210 analyser (Hitachi, Tokyo, Japan), as were kidney and heart function. The expression levels of the inflammatory cytokines tumour necrosis factor alpha (TNF-α), interleukin (IL)-2, IL-4, IL-6, and IL-10 in the serum were measured by flow cytometry using Cytometric Bead Array technology^55^.

### Faecal microbial DNA extraction, amplification and sequencing

Faecal samples were maintained at 56 °C for 30 min to inactivate the viruses. Microbial DNA was extracted using a PowerSoil Pro Kit (Qiagen, California, USA). The partial internal transcribed spacer (ITS) of fungal ribosomal DNA was amplified using the ITS3F (5′-CTTGGTCATTTAGAGGAAGTAA-3′) and ITS4R (5′-GCTGCGTTCTTCATCGATGC-3′) primers. PCR amplification was conducted with an initial denaturation at 95 °C for 90 s; 35 cycles of 95 °C for 30 s, 55 °C for 30 s, and 72 °C for 45 s; and a final extension at 72 °C for 10 min. The PCR products were purified by electrophoretic separation on a 2.0% agarose gel using the AxyPrep DNA Gel Extraction Kit (Axygen Biosciences, Union City, CA, USA) and quantified using a Quantus™ Fluorometer (Promega, USA) according to the manufacturer’s instructions. The purified amplicons were subjected to paired-end sequencing (2 × 300) using the Illumina MiSeq PE300 instrument (Illumina, San Diego, California, USA).

### Processing of sequencing data

The raw ITS gene sequencing reads were demultiplexed and quality-filtered by Trimmomatic and merged by FLASH with the following criteria: (1) 300-bp reads were truncated at any site receiving an average quality score <20 over a 50-bp sliding window, and truncated reads that were shorter than 50 bp were discarded. Reads containing ambiguous characters were also discarded. (2) Only overlapping sequences longer than 10 bp were assembled according to their overlapping sequence. The maximum permissible mismatch ratio of an overlap region was 0.2. Reads that could not be assembled were discarded. Operational taxonomic units (OTUs) with a similarity cut-off of 97% were clustered using UPARSE version 7.1 (http://drive5.com/uparse), and chimaeric sequences were identified and removed. The taxonomy of each OTU representative sequence was analysed by RDP Classifier (http://rdp.cme.msu.edu/) against the UNITE database. Alpha diversity was assessed using the Chao1 index and the Shannon index. Beta diversity was estimated by the Bray-Curtis distance and was visualized by principal coordinate analysis (PCoA). The raw sequences of the gut mycobiota were deposited into the NCBI Sequence Read Archive (SRA) database (PRJNA637034). The raw sequence data of the gut bacterial microbiota of COVID-19 patients, H1N1-infected patients and HCs were downloaded from the NCBI Sequence Read Archive (SRA) database (PRJNA636824)^19^.

### Plate counting of *Clavispora lusitaniae* CICC 32908

A loopful of *Clavispora lusitaniae* CICC 32908 cells from the slant was transferred into an Erlenmeyer flask (250 mL) containing 50 mL sterilized yeast extract peptone dextrose (YEPD) medium composed of (g/L): yeast extract 10.0; peptone 20.0; dextrose 20.0 and incubated at 30 °C and 100 rpm for 48 h. Cultures were tenfold serially diluted (from 1:10,000 to 1:1,000,000,000), and 100 μL of each dilution was plated on YEPD agar. The number of CFUs per millilitre (CFU/mL) was determined after 48 h incubation at 30 °C. Combining the number of colonies and the dispersion of colonies, we selected a dilution gradient of 10 to the eighth power. Triplicate experiments were carried out, and the mean value was calculated.

### Quantitative PCR (qPCR) primers and cycling conditions

The amount of total fungi was estimated from the quantity of ITS gene copies determined by qPCR with the ITS1f (5′-CTTGGTCATTTAGAGGAAGTAA-3′) and ITS2r (5′-GCTGCGTTCTTCATCGATGC-3′) primers^56^. qPCR was performed with a ViiA7 Real-time PCR system (Applied Biosystems, United States) using SYBR Premix Ex Taq^TM^ (RR420A; TAKARA, Tokyo, Japan). Each PCR mixture was prepared in a 10-μL final volume containing 5 μL of TB green, 0.4 μL (1 μM) of each primer, 0.2 μL of ROX reference dye, 2 μL of DNA template, and 2 μL of PCR-grade water. The reactions were hot-started at 95 °C for 30 s, followed by 40 cycles of 95 °C for 5 s and 56 °C for 34 s, followed by the melt curve stage of 95 °C for 15 s, 60 °C for 1 min, and 95 °C for 15 s. Plasmid DNA with an inserted PCR-amplified ITS gene from *Clavispora lusitaniae* CICC 32908, which was prepared similarly as described previously^57^, was used to produce a calibration curve of the ITS gene copy concentration versus cycle quantification value. Calibration curves were generated using standards with eight consecutive dilution steps (the number of fungi ranged from 1 to 10^8^ per mL). Sample DNA was analysed in duplicate without dilution.

### Statistics and reproducibility

If the clinical characteristics; haematological variables; liver, kidney and heart function; Chao1 index; and Shannon index were normally distributed based on the Kolmogorov–Smirnov test, differences between cohorts were compared using one-way ANOVA followed by the Student–Newman–Keuls method. For parameters that were not normally distributed, the Mann–Whitney *U* test was used. Analysis of similarities (ANOSIM) was performed to determine whether intergroup differences were greater than intragroup differences. The Wilcoxon rank-sum test was used to evaluate differences in the relative abundance of fungal taxa between groups, and only taxa that were significantly different in both the discovery and validation cohorts are discussed. Spearman’s rank correlation was used to analyse associations. A two-sided *P* < 0.05 was considered significant. Furthermore, absolute correlation coefficients greater than 0.4 were used as the significance thresholds of associations.

### Reporting summary

Further information on research design is available in the Nature Research Reporting Summary linked to this article.

## Supplementary information

Supplementary Information

Description of Additional Supplementary Files

Supplementary Data 1

Reporting Summary

## Data Availability

The raw sequences of the gut mycobiota were deposited into the NCBI Sequence Read Archive (SRA) database (PRJNA637034). All other data are available from the corresponding author on reasonable request.
