# Supplementary material for: Gut mycobiota alterations in patients with COVID-19 and H1N1 infections and their associations with clinical features
Source: Commun Biol. 2021 Apr 13;4:480. doi: 10.1038/s42003-021-02036-x (PMC8044104; doi:10.1038/s42003-021-02036-x)
Supplement: Supplementary file 1 — Supplementary Information [file 42003_2021_2036_MOESM1_ESM.pdf]

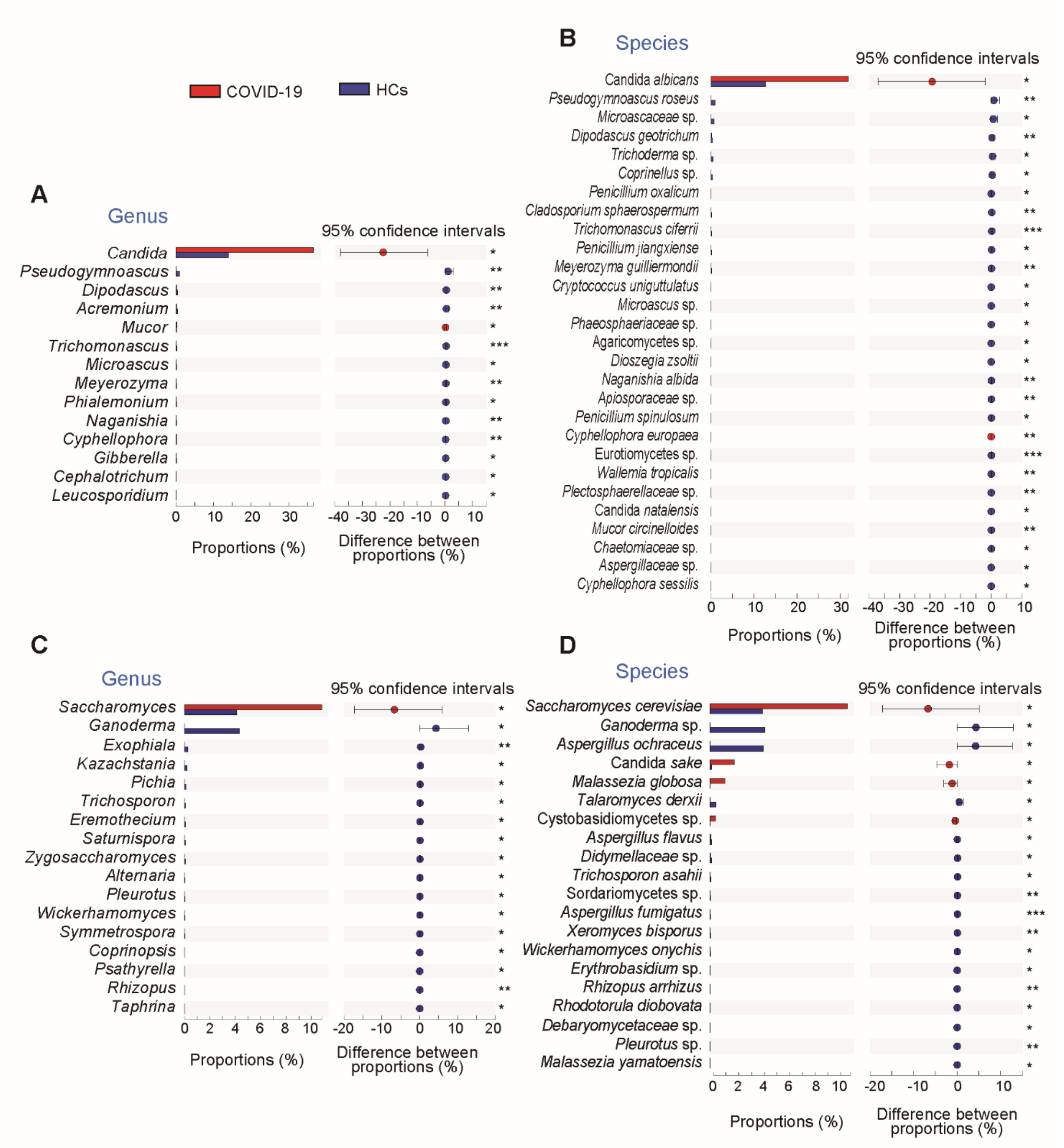

Supplementary Figure 1. Alterations in gut fungal taxa from patients with COVID-19 to HCs. (A) Genera and (B) species that were significantly altered from patients with COVID-19 compared to HCs only in the discovery cohort (COVID-19, n = 34; HCs, n = 23). (C) Genera and (D) species that were significantly altered from patients with COVID-19 to HCs only in the validation cohort (COVID-19, n = 33; HCs, n = 23). *, P < 0.05; **, P < 0.01; and ***, P < 0.001

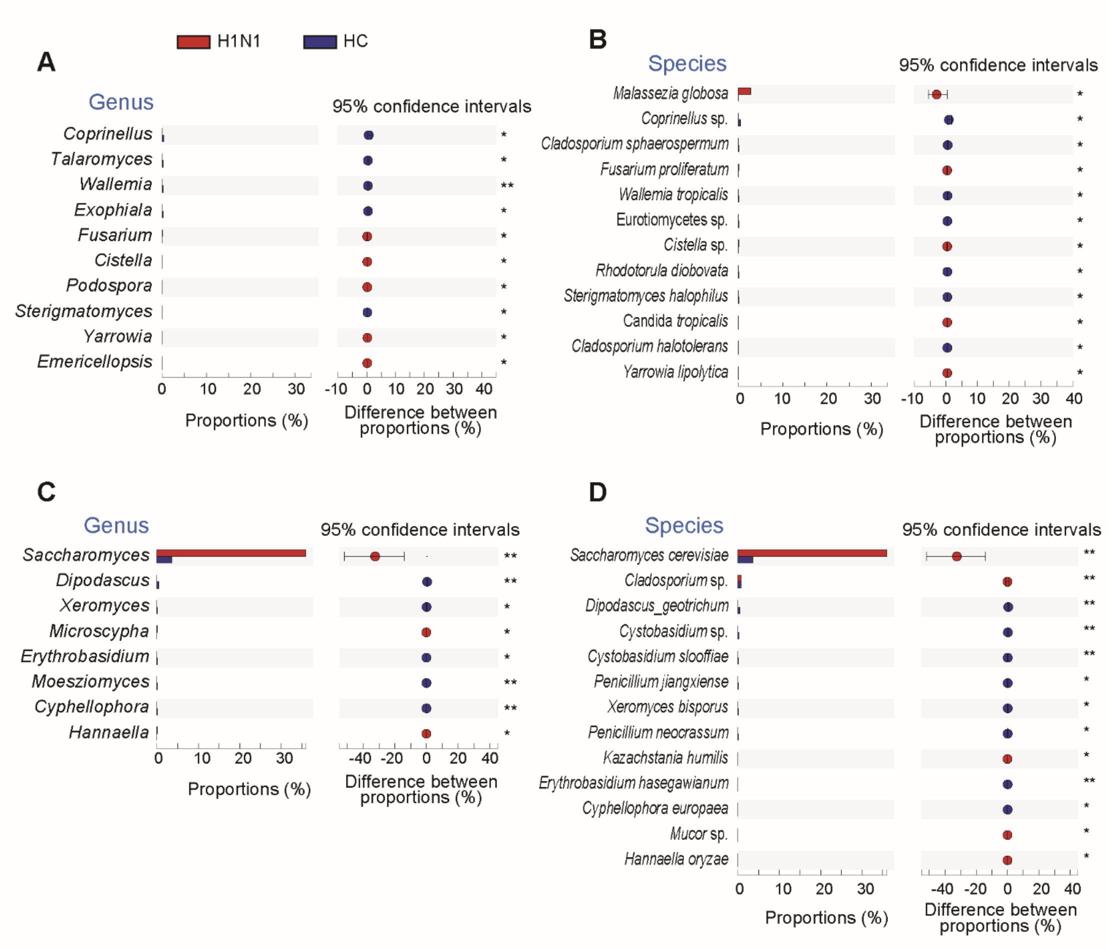

Supplementary Figure 2. Alterations in gut fungal taxa from patients with H1N1 to HCs. (A) Genera and (B) species that were significantly altered from patients with H1N1 to HCs only in the discovery cohort (H1N1, n = 20; HCs, n = 30). (C) Genera and (D) species that were significantly altered from patients with H1N1 to HCs only in the validation cohort (H1N1, n = 12; HCs, n = 16). *, P < 0.05; **, P < 0.01; and ***, P < 0.001

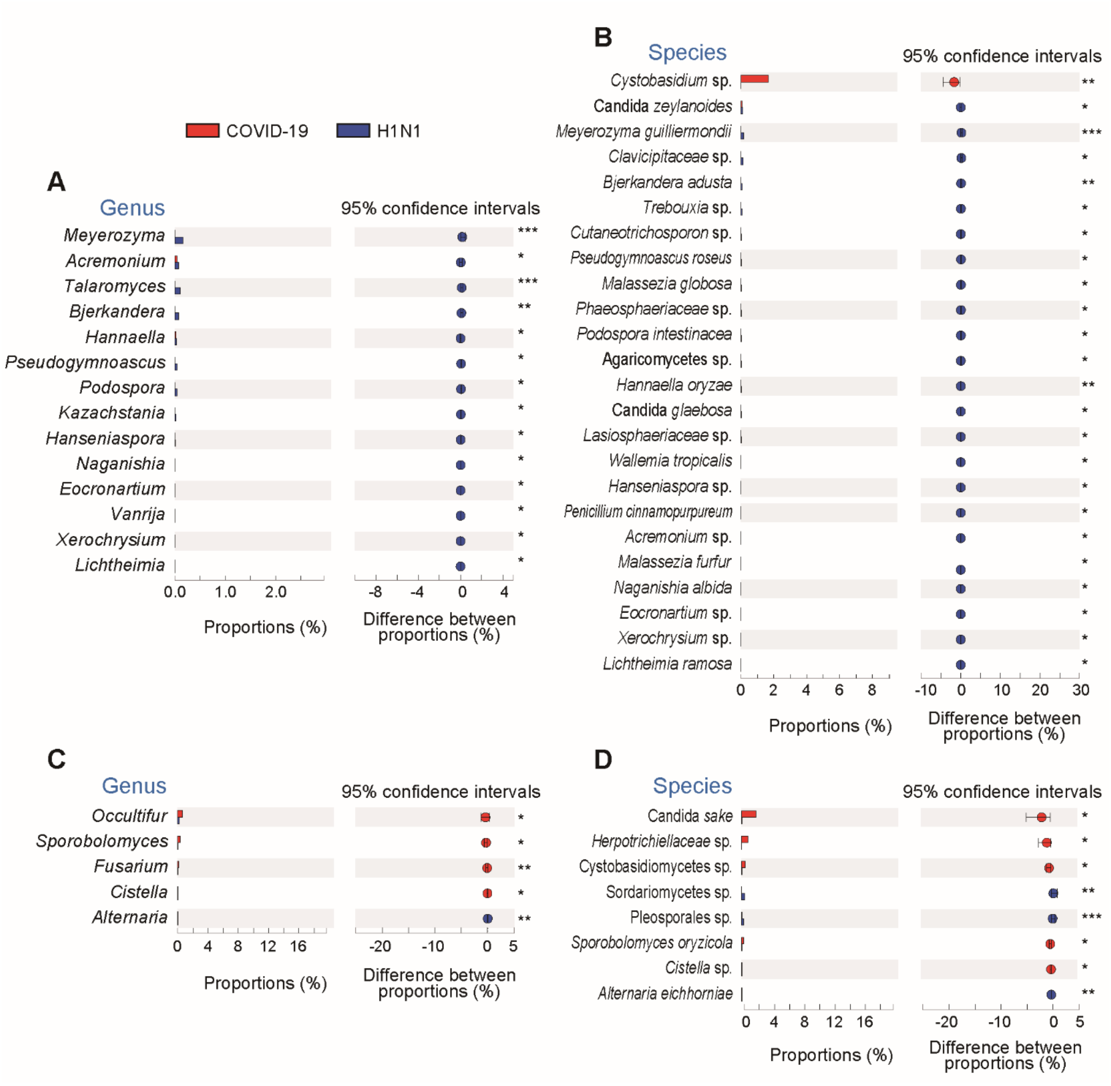

Supplementary Figure 3. Alterations in gut fungal taxa from patients with COVID-19 to patients with H1N1. (A) Genera and (B) species that were significantly altered from patients with COVID-19 to patients with H1N1 only in the discovery cohort (COVID-19, n = 34; H1N1, n = 20). (C) Genera and (D) species that were significantly altered from patients with COVID-19 to patients with H1N1 only in the validation cohort (COVID-19, n = 33; H1N1, n = 12). *, $P < 0.05$; **, $P < 0.01$; and ***, $P < 0.001$

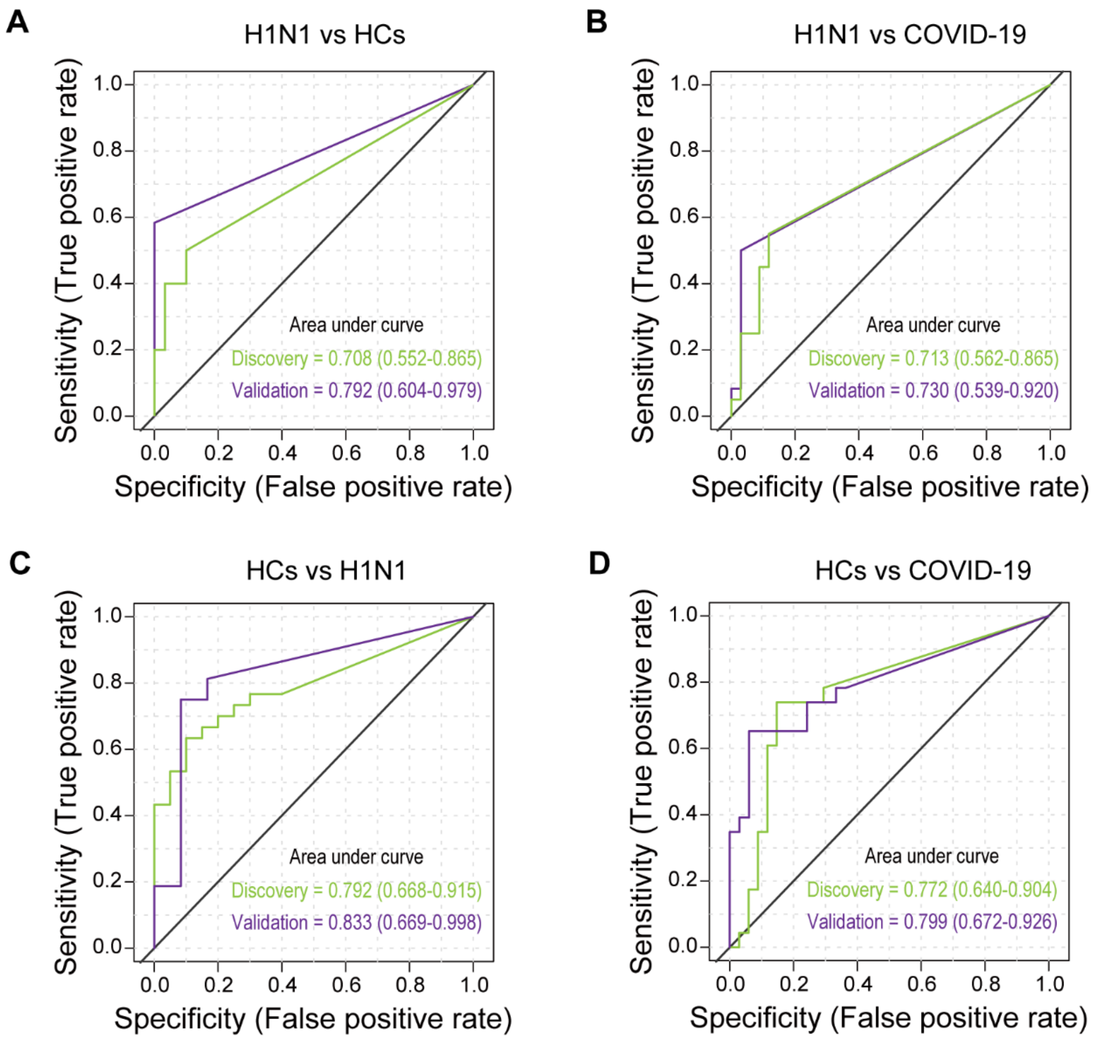

Supplementary Figure 4. Receiver operating characteristic (ROC) curves of gut fungal species that have good predicting ability. The ROC curve shows *Trebouxia decolorans* distinguished H1N1 patients from (A) HCs or (B) COVID-19 patients in both the discovery cohort and the validation cohort. The ROC curve shows *T Penicillium polonicum* distinguished HCs from (C) H1N1 patients or (D) COVID-19 patients in both the discovery cohort and the validation cohort.
