# Supplementary material for: Gut mycobiota alterations in patients with COVID-19 and H1N1 infections and their associations with clinical features
Source: Commun Biol. 2021 Apr 13;4:480. doi: 10.1038/s42003-021-02036-x (PMC8044104; doi:10.1038/s42003-021-02036-x)
Supplement: Supplementary file 2 — Description of Additional Supplementary Files [file 42003_2021_2036_MOESM2_ESM.pdf]

Description of Additional Supplementary Files

File name: Supplementary Data 1

Description: All source data underlying the graphs and charts.
