# Supplementary material for: Gut mycobiota alterations in patients with COVID-19 and H1N1 infections and their associations with clinical features
Source: Commun Biol. 2021 Apr 13;4:480. doi: 10.1038/s42003-021-02036-x (PMC8044104; doi:10.1038/s42003-021-02036-x)
Supplement: Supplementary file 4 — Reporting Summary [file 42003_2021_2036_MOESM4_ESM.pdf]

nature research

Corresponding author(s): Lanjuan Li

Last updated by author(s): Jan 12, 2021

Reporting Summary

Nature Research wishes to improve the reproducibility of the work that we publish. This form provides structure for consistency and transparency in reporting. For further information on Nature Research policies, see our Editorial Policies and the Editorial Policy Checklist.

Statistics

For all statistical analyses, confirm that the following items are present in the figure legend, table legend, main text, or Methods section.

n/a	Confirmed	
☐	☒	The exact sample size (*n*) for each experimental group/condition, given as a discrete number and unit of measurement
☐	☒	A statement on whether measurements were taken from distinct samples or whether the same sample was measured repeatedly
☐	☒	The statistical test(s) used AND whether they are one- or two-sided<br>*Only common tests should be described solely by name; describe more complex techniques in the Methods section.*
☐	☒	A description of all covariates tested
☐	☒	A description of any assumptions or corrections, such as tests of normality and adjustment for multiple comparisons
☐	☒	A full description of the statistical parameters including central tendency (e.g. means) or other basic estimates (e.g. regression coefficient) AND variation (e.g. standard deviation) or associated estimates of uncertainty (e.g. confidence intervals)
☒	☐	For null hypothesis testing, the test statistic (e.g. *F*, *t*, *r*) with confidence intervals, effect sizes, degrees of freedom and *P* value noted<br>*Give P values as exact values whenever suitable.*
☒	☐	For Bayesian analysis, information on the choice of priors and Markov chain Monte Carlo settings
☒	☐	For hierarchical and complex designs, identification of the appropriate level for tests and full reporting of outcomes
☒	☐	Estimates of effect sizes (e.g. Cohen's *d*, Pearson's *r*), indicating how they were calculated

Our web collection on statistics for biologists contains articles on many of the points above.

Software and code

Policy information about availability of computer code

Data collection	No
Data analysis	Usearch (vsesion 7.0); Unite (Release 7.2); RDP Classifier (version 2.2); mothur (version v.1.30.1);Qiime2;R Programming Language (PCoA, stats, MetagenomeSeq, andomForest package; plotROC package); Networkx; PICRUSt2

For manuscripts utilizing custom algorithms or software that are central to the research but not yet described in published literature, software must be made available to editors and reviewers. We strongly encourage code deposition in a community repository (e.g. GitHub). See the Nature Research guidelines for submitting code & software for further information.

Data

Policy information about availability of data

All manuscripts must include a data availability statement. This statement should provide the following information, where applicable:

- Accession codes, unique identifiers, or web links for publicly available datasets
- A list of figures that have associated raw data
- A description of any restrictions on data availability

The raw sequences of the gut mycobiota were deposited into the NCBI Sequence Read Archive (SRA) database (PRJNA637034). All other data are available from the corresponding author on reasonable request.

Field-specific reporting

Please select the one below that is the best fit for your research. If you are not sure, read the appropriate sections before making your selection.

☒ Life sciences ☐ Behavioural & social sciences ☐ Ecological, evolutionary & environmental sciences

For a reference copy of the document with all sections, see nature.com/documents/nr-reporting-summary-flat.pdf

Life sciences study design

All studies must disclose on these points even when the disclosure is negative.

Sample size	No sample-size calculation was performed. Because the COVID-19 had been well controlled in Zhejiang Province, China, we took all eligible patient samples from our hospital that is the only designated hospital of COVID-19 in Hangzhou city of Zhejiang province during the study period. There were 184 samples for COVID-19, H1N1 patients and healthy controls. We tried our best to set discovery and validation cohort to ensure the accuracy of our results.
Data exclusions	No data were excluded from the analyses.
Replication	All attempts a replication were successful.
Randomization	Samples and participants were randomly allocated into experiment groups.
Blinding	n/a; During data collection and/or analysis, investigators need to know the group allocation to accurately conduct their research.

Reporting for specific materials, systems and methods

We require information from authors about some types of materials, experimental systems and methods used in many studies. Here, indicate whether each material, system or method listed is relevant to your study. If you are not sure if a list item applies to your research, read the appropriate section before selecting a response.

Materials & experimental systems

n/a	Involved in the study	
☒	☐	Antibodies
☒	☐	Eukaryotic cell lines
☒	☐	Palaeontology and archaeology
☒	☐	Animals and other organisms
☐	☒	Human research participants
☒	☐	Clinical data
☒	☐	Dual use research of concern

Methods

n/a	Involved in the study	
☒	☐	ChIP-seq
☒	☐	Flow cytometry
☒	☐	MRI-based neuroimaging

Human research participants

Policy information about studies involving human research participants

Population characteristics	Nearly 54% of COVID-19 patients and 68% of H1N1 patients had severe disease. The most common underlying diseases were hypertension, diabetes mellitus and liver diseases, while the most common symptoms were fever, cough and diarrhoea in both COVID-19 and H1N1 patients. All patients with H1N1 or COVID-19 were treated with antiviral drugs; some were treated with glucocorticoids, but no patients received antifungal drugs, probiotics or prebiotics. No patients died, but the median hospital stay of COVID-19 patients was approximately 16 days, which was much higher than the median hospital stay of 7 days of H1N1 patients.<br>The absolute lymphocyte count and neutrophil count in peripheral blood were not significantly different between COVID-19 patients and H1N1 patients, but the lymphocyte count was lower and the neutrophil count was higher in both patient groups than in HCs. C-reactive protein (CRP) was significantly higher in COVID-19 patients and H1N1 patients than in HCs, while procalcitonin was higher in H1N1 patients than in COVID-19 patients and HCs. Additionally, compared with HCs, both H1N1 patients and COVID-19 patients had significant increases in IL-2, IL-6, and IL-10. IL-4 and TNF-α were higher in COVID-19 patients than H1N1 patients and HCs. Compared with HCs, ALB decreased and ALT and GGT increased in both H1N1 patients and COVID-19 patients. Interestingly, AST increased in only H1N1 patients, and was even higher than that in COVID-19 patients. ALP decreased in only COVID-19 patients compared to HCs. Compared with HCs, the absolute erythrocyte count in peripheral blood decreased in both COVID-19 patients and H1N1 patients, while haemoglobin decreased in only the COVID-19 group.
Recruitment	In COVID-19 patients hospitalized from January to February 2020, and H1N1 patients hospitalized from January 2018 to January 2020, those did not using antibiotics or antifungal agents were recruited. Therefore, the patient recruitment can be considered as random. The health controls came from the physical examination center of our hospital. The health controls

	did not have a history of major diseases or suffer from other diseases at the time of sampling. Meanwhile, according to the data of their physical examination center, we can confirm their health status. All healthy controls were age- and gender-matched with patients.
Ethics oversight	the ethics committee of the first affiliated hospital, college of medicine First Affiliated Hospital, College of Medicine, Zhejiang University (Process Nos. IIT2020-136 and 2018-447).

Note that full information on the approval of the study protocol must also be provided in the manuscript.
